# Toripalimab treatment of bladder-preserving therapy for locally advanced bladder cancer: a case report

**DOI:** 10.3389/fonc.2025.1581452

**Published:** 2025-06-17

**Authors:** Zhiyun Yang, Yidao Liu, Ji Li, Xingsheng Wang, Jun Pan, Jiafu Jiang, Xingming Zhang

**Affiliations:** ^1^ Department of Urology, Yunnan Dehong People’s Hospital, Dehong, China; ^2^ Department of Anesthesia, Yunnan Dehong People’s Hospital, Dehong, China; ^3^ Department of Urology, West China Hospital, Sichuan University, Chendu, China

**Keywords:** muscle-invasive bladder cancer, bladder-preserving therapy, immune therapy, Toripalimab, PD-L1, CD8+ T lymphocytes

## Abstract

Neoadjuvant chemotherapy and radical cystectomy is the standard treatment for muscle invasive bladder cancer. For patients who are intolerant or unwilling to receive radical surgery and chemotherapy, the use of immune therapy combined with bladder preservation treatment has gradually become a viable treatment option. In this article, We aimed to present a case of cT2N0M0 Bladder cancer patient who was intolerant to chemotherapy and was treated with Toripalimab as a single-agent bladder preservation therapy for three cycles. After 15 months of treatment, a clinical complete response(cCR) was achieved, thus retaining the bladder. In this report, the safety profile of Toripalimab was favorable, with no severe or uncontrollable adverse events. The patient’s pathological IHC showed PD-L1 negative (TPS <1%, CPS <1%) and did not undergo mTURBT, which is a rare phenomenon. Our report suggests that Patients with PD-L1 negative but CD8+ T cell positive tumor infiltration can also benefit from PD-L1 inhibitor treatment and even achieve cCR to preserve the bladder.

## 1 Introduction

Bladder cancer is one of the most common malignancies in the urinary system, ranking as the 10th most prevalent new malignancy in the world (1). Based on the depth of tumor invasion, it can be classified into non-muscle-invasive bladder cancer (NMIBC) and muscle-invasive bladder cancer (MIBC). MIBC accounts for approximately 25% of all bladder cancers and has a poor clinical prognosis (2). The current standard treatment for MIBC involves neoadjuvant chemotherapy, radical cystectomy (RC), and pelvic lymph node dissection (PLND) (2, 3). However, RC is a highly morbid procedure, especially for elderly patients with poor performance status, with a perioperative complication and 90-d mortality rates of 47.2% and 9.35-10.6%, respectively (4). Studies have shown that approximately 49% of MIBC patients opt for bladder preservation therapies (BPT) due to decreased post-surgical quality of life and reluctance to change the mode of urination (5). Therefore, BPT is an important treatment in clinical practice, especialy in those with high risk of perioperative complication and mortality, or unwilling to receive RC.

The trimodality therapy (TMT) is currently the most evidence-based bladder-preserving treatment regimen with the strongest backing from clinical studies. This regimen includes three components: maximal transurethral resection of bladder tumor (mTURBT), systemic chemotherapy, and local radiation therapy. In recent years, immune therapy has achieved satisfactory results in clinical trials for tumor treatment, and it has gradually become a second-line or even first-line treatment option for bladder cancer (3). For MIBC patients who are intolerant or unwilling to receive RC, the use of immune therapy combined with bladder preservation treatment has gradually become a viable treatment option. In this article, we report on a case of a cT2N0M0 Bladder cancer patient who was intolerant to chemotherapy and was treated with toripalimab as a single-agent bladder preservation therapy for three cycles. After 15 months of treatment, a clinical complete response(cCR) was achieved.

## 2 Case report

### 2.1 Medical history and diagnosis

**Figure 1:**
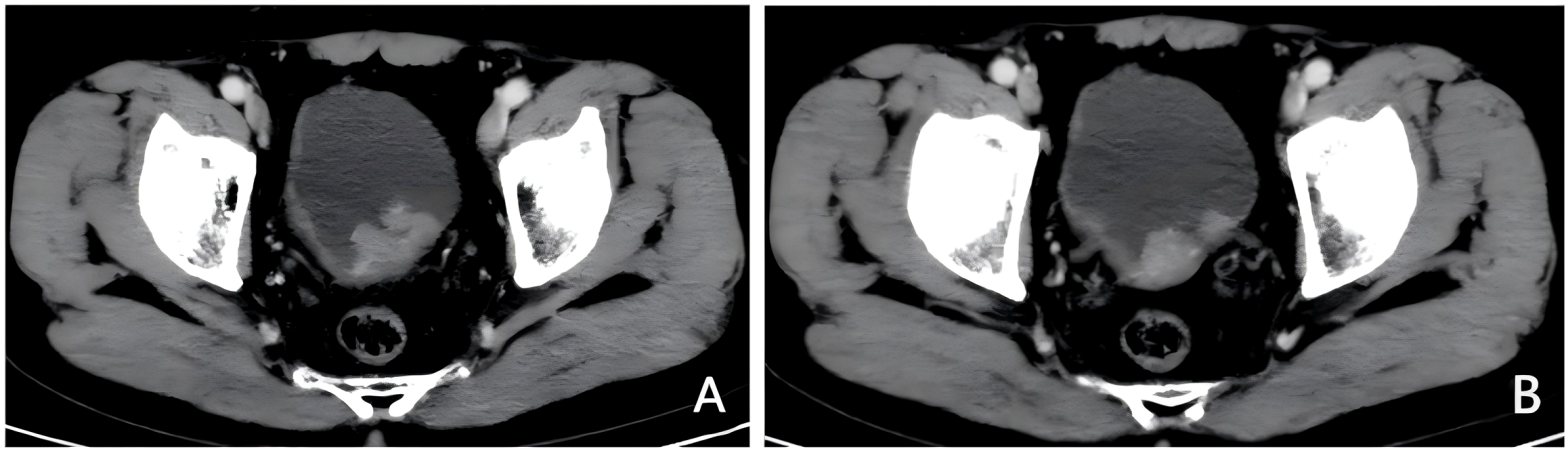
Imaging examination before treatment. **(A, B)** Pre-treatment enhanced CT, bladder posterior wall thickening with multiple irregular masses, approximately 4.4*2.6cm in size.

**Figure 2:**
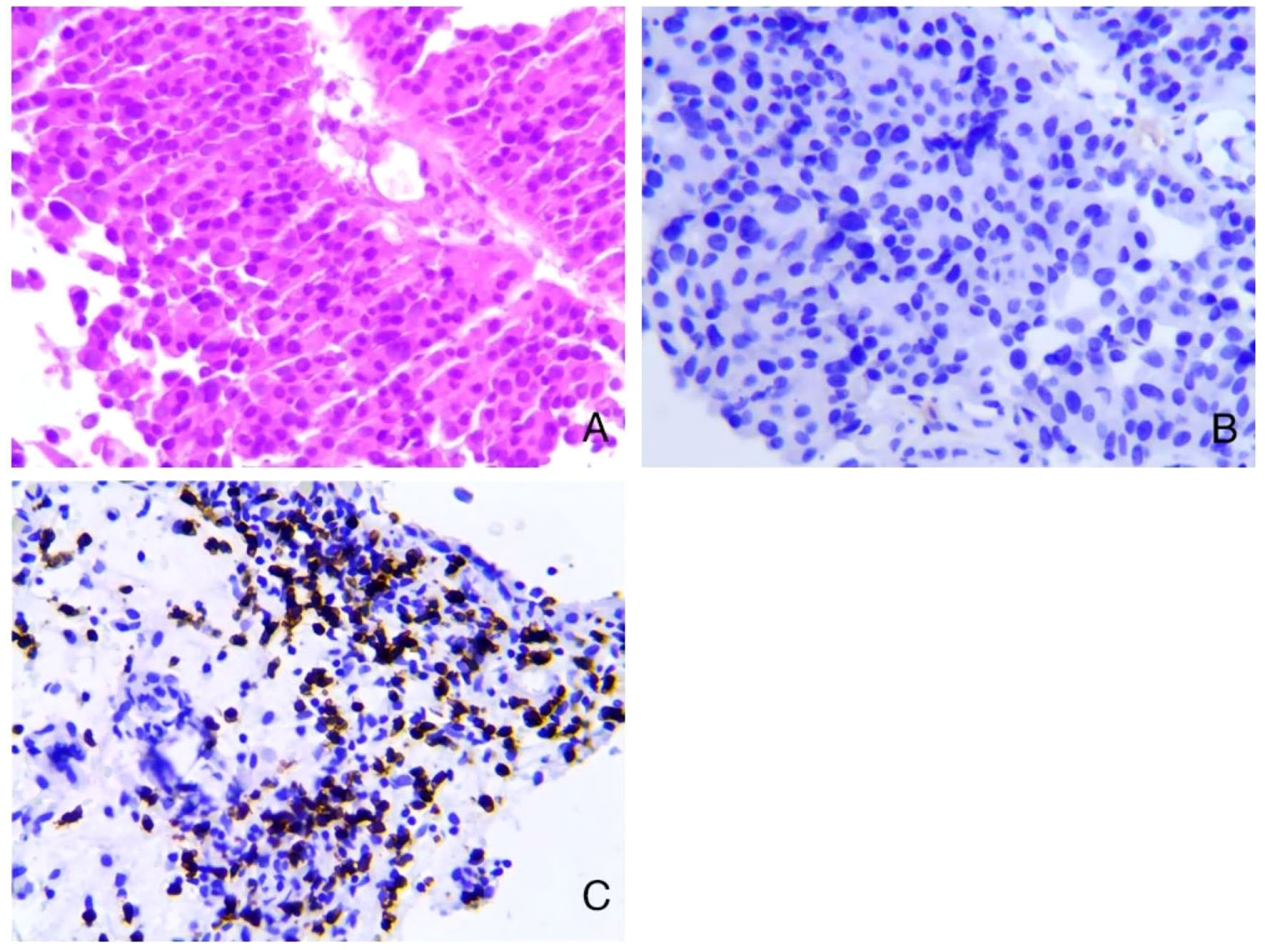
Pathological results before treatment. **(A)** Low-grade papillary urothelial carcinoma (HE staining, 200X). **(B)** PD-L1 negative control (E1L3N, 200X). **(C)** CD8+ T 60% positive (IHC, 200X).

A 71-year-old male patient, with a history of smoking but currently quit, has no family history of cancer, a history of hypertension, and is without a history of diabetes or heart disease. He presented to our hospital with intermittent gross hematuria lasting over a year on April 30th, 2023. Initial physical examination upon admission showed no significant abnormalities.Urine routine examination revealed a red blood cell count of 211.2/ul; serum creatinine was 86umol/L; peripheral blood lymphocyte examination showed a percentage of 30.3% for the helper/inducer T-lymphocyte CD4+ and 53.1% for the suppressor/T-toxic T-lymphocyte CD8+. ECG, cardiac ultrasound, and head and lung CT scans showed no significant abnormalities. Enhanced abdominal CT (**Figures 1A, B**) revealed thickening of the posterior bladder wall with multiple irregular masses, approximately 4.4*2.6cm in size, suggestive of bladder malignancy. There were also multiple kidney stones and multiple liver cysts. Cystoscopy showed multiple cauliflower-like lesions on the left posterior bladder wall, with the largest tumor measuring approximately 4.5cm, accompanied by surface necrosis and bleeding. The left ureteral opening could not be explored. Pathological biopsy of the tumor revealed low-grade papillary urothelial carcinoma. Immunohistochemical (IHC)staining (**Figures 2A–C**) showed PD-L1 (TPS<1%, CPS<1), CD8+ T (60% positive). The clinical diagnosis was bladder cancer at stage T2N0M0.

### 2.2 Treatment and results

The patient was presented with the following treatment options during initial counseling: 1. Maximal TURBT followed by adjuvant therapy, 2. Radical cystectomy (RC) or 3. Neoadjuvant therapy followed by reassessment for bladder preservation (with maximal TURBT or RC based on response). The patient initially opted for neoadjuvant therapy followed by radical cystectomy for bladder cancer. Therefore, the patient’s initial plan was to undergo GC protocol neoadjuvant chemotherapy followed by radical cystectomy. The first cycle of GC protocol chemotherapy began on May 2nd, 2023. However, due to severe gastrointestinal reactions including frequent vomiting and loss of appetite after cisplatin use (Grade 3 according to CTCAE 5.0), the patient’s chemotherapy was discontinued. We recommended that the patient undergo radical surgery or switch to neoadjuvant immunotherapy. After discussing the treatment plan and associated risks with the patient, the patient opted for neoadjuvant immunotherapy. On May 12th, the neoadjuvant treatment plan was changed to immune therapy with Toripalimab (produced by Shanghai Junshi Biosciences Pharmaceutical Co., Ltd., with a drug approval number of S20191003). The treatment involved a 240mg dose of Toripalimab diluted in 100ml of normal saline, administered as a slow intravenous infusion once every 3 weeks. The patient showed no special adverse reactions during the observation period.

**Figure 3:**
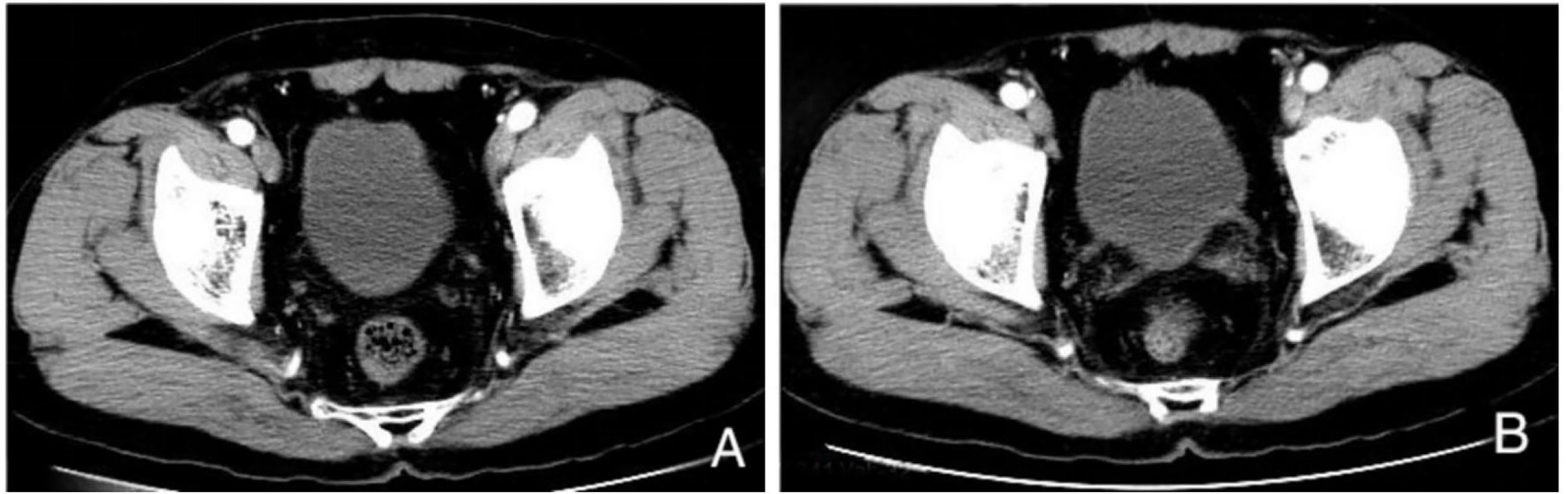
Enhanced CT examination after 15 months of immunotherapy. **(A, B)** Multiple masses on the posterior wall of the bladder have disappeared compared to previous images.

**Figure 4:**
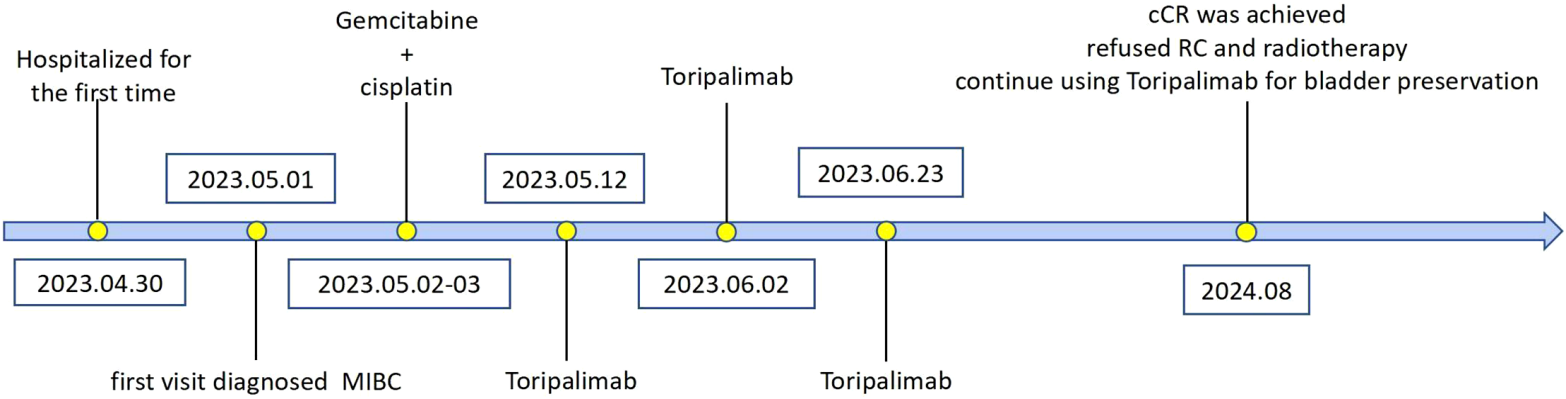
Chronologic evolution of treatments among our patient.

After receiving Toripalimab for three cycles, the patient’s hematuria symptoms disappeared, and the patient did not return to the hospital for further treatment. The patient returned to our hospital for a review on August 4th, 2024. A contrast-enhanced CT scan of the chest and abdomen (**Figures 3A, B**) showed that multiple masses on the posterior wall of the bladder had disappeared compared to previous images. On August 6th, under anesthesia, the patient underwent cystoscopy and multiple mucosal biopsies, which revealed that the bladder tumor had disappeared. The pathological results showed chronic inflammation of the bladder mucosa with focal calcification. The patient achieved cCR after immunotherapy. We recommended that the patient could subsequently undergo either radical surgery or bladder-preserving therapy. Given the satisfactory response to immunotherapy and the patient’s strong preference for maintaining a high quality of life, he opted for bladder-preserving treatment. It was recommended that the patient undergo radiotherapy combined with immunotherapy. However, the patient was unable to accept radiotherapy. The treatment plan was therefore adjusted to continue using Toripalimab for bladder preservation, with cystoscopy and CT scans to be performed every three months for follow-up. Evolution of these different treatments is represented in **Figure 4**.

## 3 Discussion

MIBC is a fatal malignancy with a 5-year survival rate of 50% (6). The currently recommended standard treatment by major guidelines involves neoadjuvant chemotherapy based on cisplatin, followed by RC. Due to the decreased quality of life after RC surgery, some patients have a strong desire for BPT. According to data from the National Cancer Database of the United States, out of 28,691 patients diagnosed with MIBC between 2004 and 2008, only less than half of the MIBC patients received RC, while nearly half of them opted for BPT treatment (5). A crucial focus in the implementation of BPT is whether it can achieve a survival rate comparable to or matching the current RC scheme. Studies have shown that there is no significant difference in long-term survival between the TMT scheme and RC (7, 8). Hu’s study suggests that patients who achieve pathological complete response after neoadjuvant treatments plus maximal transurethral resection of the bladder tumor may be safe to receive bladder preservation therapy (9). These studies provide evidence support for the clinical application of comprehensive bladder-preserving treatment, at the same time, it also emphasizes the important role of maximum TURBT in bladder preservation therapy.

On the other hand, the proportion of neoadjuvant treatment is continuously increasing in patients undergoing RC (10). Approximately 10%-40% of patients can achieve cCR after neoadjuvant chemotherapy. Studies have shown that the 5-year survival rate of patients with cCR who proceed with RC can exceed 90% (11). However, some cCR patients, whose tumors are no longer visible, refuse to proceed with the original plan of RC and opt for bladder-preserving treatment. Mazza found that even with close follow-up after cCR from neoadjuvant chemotherapy, the 5-year survival rate can still reach 86%, and the recurrence-free survival rate exceeds 60% (12). Preoperative neoadjuvant treatment with immune drugs alone or in combination with other agents increases the pathological complete response (pCR) rate and pathological downstaging rate in MIBC. Compared to chemotherapy, most immune treatment-related adverse reactions are grade 1-2, providing a safer and more effective treatment option for MIBC patients who are elderly, weak, or intolerant to chemotherapy. To benefit chemotherapy-intolerant MIBC patients from neoadjuvant treatment, a multicenter phase II clinical trial on Atezolizumab (ABACUS study) showed that the overall pCR rate was 31%, with a 1-year recurrence-free survival (RFS) rate of 79%. In PD-L1-positive patients, the pCR rate reached 37%, with 54% of patients having a pathological stage downgraded to NMIBC, and a 1-year RFS rate of 75%. The expression of PD-LI does not have a statistically significant difference in prognosis. Additionally, researchers found that high expression of CD8 before treatment is associated with 40% CRP and an 85% 1-year survival rate in the CD8+ population (13). Another single-arm phase II clinical trial on Pembrolizumab (PURE-01 study) showed that the overall pCR rate was 37%, with 55% of patients achieving tumor downstaging. In multivariate logistic regression, PD-LI CPS is an independent risk factor for achieving PT0 and PT ≤ 1 after immunotherapy for MIBC (14). Additionally, it is noteworthy that in this study, two patients refused RC after the achievement of a radiological complete response. They did not receive further treatment at +6 and +3 months of follow-up, yet showed no disease recurrence or progression. This observation suggests the potential feasibility of bladder-preserving therapy for bladder cancer patients who achieve pCR or cCR following PD-L1 therapy.

Our study reports on a case of a MIBC patient who was intolerant to chemotherapy and was treated with a single-agent immune checkpoint inhibitor for three cycles, the safety profile of Toripalimab was favorable, with no grade 3-4 adverse events. After 15 months of treatment, the patient achieved cCR with satisfactory treatment results and was then switched to bladder-preserving treatment. The patient’s pathological IHC showed PD-L1 negative (TPS <1%, CPS <1%) and did not undergo mTURBT, which is a rare phenomenon. There are several possibilities that may explain this phenomenon:

First, various solid tumors can suppress the tumor microenvironment and avoid being lysed by lymphocytes through the expression of PD-L1 binding to PD1 on the surface of T cells. However, current PD-L1 detection based on IHC has problems not only in determining which tumor tissues respond to immune therapy based on anti-PD-1/PD-L1 but also in determining which individual patients may benefit from treatment (15). Hu’s study suggests that there was no significant difference between response and resistance samples regarding the infiltration level of tumor-associated immune cells and the expression of PD-L1 and PD-1 (9). PD-L1 as a biomarker remains controversial mainly because it cannot accurately predict individual treatment responses (15, 16).

Secondly, CD8+ T cells (cytotoxic T lymphocytes, CTL) are the preferred immune cells targeting tumors (17). They kill tumor cells by releasing granules or inducing FasL-mediated apoptosis and release interferon-γ (IFNγ) and tumor necrosis factor α (TNFα) to induce tumor cell cytotoxicity (18). Naito’s study showed that the aggregation of CD8+ T cells in colon cancer predicts improved patient survival (19). Follow-up studies on ovarian cancer, melanoma, and colon cancer have further shown that the proportion and distribution of tumor-infiltrating CD8+ T cells and T_reg_ cells are critical determinants of prognosis (20–23). Robert’s study suggests that the type and density of tumor-infiltrating lymphocytes are better predictors of patient outcome compared to pathological stage and oncogene expression (24). Recent study shows that the number of T/NK cells of neoadjuvant ICB(NICB) resistance group was smaller than that of NICB response group. Especially, the proportions of CD8+ T cells and NK cells were lower in NICB resistance group, which highlighted the critical role of CD8+ T cells and NK cells for NICB response (9). In addition, lower clinical stage (T2N0M0), pure urothelial carcinoma (UC) histology, cell cycle subtype were, combine immunotherapy with targeted therapy significantly associated with higher pathological response rates (9, 25).

Although the case reported in this study was negative for PD-L1 expression, it exhibited strong CD8+ T lymphocyte infiltration, the clinical stage was T2N0M0, and the tumor was a pure UC. These factors may be the primary reasons for the satisfactory therapeutic response to PD-1 inhibitor in this case.

However, although this patient achieved satisfactory therapeutic outcomes with neoadjuvant treatment, clinical practice demonstrates that not all patients respond equally to this regimen. A significant proportion of patients fail to derive clinical benefit from such treatment approaches. Therefore, when making therapeutic decisions, clinicians should carefully consider cost-effectiveness implications, particularly given the substantial financial burden associated with immunotherapy. As highlighted by Contieri, the high cost of these treatments often not covered by health insurance in various countries and regions poses considerable challenges for patients. More comprehensive cost-effectiveness analyses are warranted to establish optimal and sustainable treatment strategies for future clinical applications (26).

## 4 Conclusion

In summary, for MIBC patients who are intolerant to chemotherapy, immunotherapy for bladder preservation is a viable treatment option. Patients with PD-L1 negative but CD8+ T cell positive tumor infiltration can also benefit from PD-L1 inhibitor treatment and even achieve cCR to preserve the bladder. Although this treatment regimen demonstrated good safety and effectiveness in this case, as a single-case report, we cannot rule out accidental and individual differences. Further studies are needed to explore the efficacy and indications of immunotherapy in bladder-preserving treatment for MIBC, providing better treatment options for patients.

## Data Availability

The original contributions presented in the study are included in the article/supplementary material. Further inquiries can be directed to the corresponding author.
